# Comprehensive RNA-Seq Data Analysis Identifies Key mRNAs and lncRNAs in Atrial Fibrillation

**DOI:** 10.3389/fgene.2019.00908

**Published:** 2019-10-02

**Authors:** Dong-Mei Wu, Zheng-Kun Zhou, Shao-Hua Fan, Zi-Hui Zheng, Xin Wen, Xin-Rui Han, Shan Wang, Yong-Jian Wang, Zi-Feng Zhang, Qun Shan, Meng-Qiu Li, Bin Hu, Jun Lu, Gui-Quan Chen, Xiao-Wu Hong, Yuan-Lin Zheng

**Affiliations:** ^1^Key Laboratory for Biotechnology on Medicinal Plants of Jiangsu Province, School of Life Science, Jiangsu Normal University, Xuzhou, China; ^2^College of Health Sciences, Jiangsu Normal University, Xuzhou, China; ^3^State Key Laboratory Cultivation Base For TCM Quality and Efficacy, School of Medicine and Life Science, Nanjing University of Chinese Medicine, Nanjing, China; ^4^State Key Laboratory of Pharmaceutical Biotechnology, MOE Key Laboratory of Model Animal for Disease Study, Model Animal Research Center, Nanjing University, Nanjing, China; ^5^Department of Immunology, School of Basic Medical Sciences, Fudan University, Shanghai, China

**Keywords:** long non-coding RNAs, atrial fibrillation, RNA-Seq, genes, protein coding genes

## Abstract

Long non-coding RNAs (lncRNAs) are an emerging class of RNA species that may play a critical regulatory role in gene expression. However, the association between lncRNAs and atrial fibrillation (AF) is still not fully understood. In this study, we used RNA sequencing data to identify and quantify the both protein coding genes (PCGs) and lncRNAs. The high enrichment of these up-regulated genes in biological functions concerning response to virus and inflammatory response suggested that chronic viral infection may lead to activated inflammatory pathways, thereby alter the electrophysiology, structure, and autonomic remodeling of the atria. In contrast, the downregulated GO terms were related to the response to saccharides. To identify key lncRNAs involved in AF, we predicted lncRNAs regulating expression of the adjacent PCGs, and characterized biological function of the dysregulated lncRNAs. We found that two lncRNAs, ETF1P2, and AP001053.11, could interact with protein-coding genes (PCGs), which were implicated in AF. In conclusion, we identified key PCGs and lncRNAs, which may be implicated in AF, which not only improves our understanding of the roles of lncRNAs in AF, but also provides potentially functional lncRNAs for AF researchers.

## Introduction

Atrial fibrillation (AF), one of the most common serious arrhythmia worldwide, whose extreme complications such as heart failure and embolic stroke are often of high risks and associated with increasing morbidity and mortality (Conen et al., 2011). Atrial remodeling, both electrical and structural, are important characteristics in AF (Li et al., 2017; Allessie et al., 2002). AF could bring permanent changes such as enlarged left and right atrial size. Moreover, increasing left atrial volume has been stated as a risk factor of cardioembolic stroke, and it is critical to interpret the mechanism behind this to improve the stroke prevention strategy.

The etiology of AF has not been fully elucidated as a varying range of factors would contribute to AF, such as family history, unhealthy lifestyle, high blood pressure and other diseases (Shi et al., 2013). With the development of next-generation sequencing technologies, non-coding RNAs (ncRNAs) emerged as the epicenter for researchers to further explore the genetic cause behind AF. ncRNAs, which can be subdivided into small ncRNAs (< 200 nt) and long ncRNAs (lncRNAs), are not translated in proteins, but some of them are capable of regulating various cellular processes such as the expression of certain genes. Evidences have verified that many lncRNAs, often generated from transcriptional units, play a critical role in several cardiovascular diseases (Su et al., 2018), and it is of great importance to survey how they function in AF and how they are connected with atrial remodeling. Several researches are conducted to explore how lncRNAs acted as regulators in atrial electrical remodeling, revealing that TCONS_00075467 may help decrease AF vulnerability through suppressing the electrical remodeling (Li et al., 2017). Recent reports have also unveiled that lncRNAs can act as modulators of miRNA levels in various cardiac diseases (Greco et al., 2018). Also, inflammation and AF are confirmed to have a close relationship. Abundant inflammatory markers and higher ratios of neutrophil and lymphocyte are often observed in patients with AF (Hu et al., 2015), and AF subsequently triggers more inflammatory response, which in turns results in worsened conditions. Exploring active lncRNAs in inflammation would shed light on the prevention, diagnosis, and therapeutic strategies of AF, and help elucidate underlying mechanisms.

In the present study, we identified differentially expressed lncRNAs and mRNAs in patients with AF and predicted lncRNA function in a co-expression-based manner. Prediction of cis-acting lncRNAs and functional annotation of dysregulated lncRNAs screened out some critical lncRNAs implicated in AF, which not only improves our understanding of the roles of lncRNAs in AF, but also provides potentially functional lncRNAs for AF researchers. In addition, as evidence proves that a variety of inflammation-associated cytokines and chemokines are involved in the pathogenesis of AF (Schnabel et al., 2009), we further investigate whether our findings are related to cytokines and chemokines in certain aspects.

## Materials and Methods

### Data Collection

We collected RNA sequencing data of 6 cases with AF and 6 controls from Sequence Read Archive (SRA, https://www.ncbi.nlm.nih.gov/sra) database (Leinonen et al., 2011) with an accession number SRP093226, which was provided by previous study (Yu et al., 2017). We uncompressed the SRA files by fastq-dump with the option –*split-files*, which generated two paired fastq files.

### Read Mapping and Gene Expression Quantification

For each sample, we first mapped the RNA-seq reads to UCSC hg19 human reference genome (www.genome.ucsc.edu) using hisat2 (Kim et al., 2015), and then sorted the SAM files by samtools. With the gene annotation from GENCODE v19 (Harrow et al., 2012), the gene expression was estimated by the StringTie (Pertea et al., 2015) and ballgown pipeline. We considered genes with biotypes, including ‘processed_transcript’, ‘pseudogene’, ‘lincRNA’, ‘3prime_overlapping_ncrna’, ‘antisense’, ‘sense_intronic’, and ‘sense_overlapping’, as lncRNAs.

### Differential Expression Analysis

The FPKM-based expression was used to identify differentially expressed genes. The gene expression values were first transformed to log_2_ (FPKM + 1), and then tested for differential expression by t test. The differentially expressed genes were identified with the thresholds of *P*-value <0.05 and fold change >2 or <1/2.

### Gene Ontology Enrichment Analysis

The Gene Ontology (GO) enrichment analysis was implemented in R with clusterProfiler package (Yu et al., 2012), which used overrepresentation enrichment analysis (ORA) to identify enriched GO terms. The GO terms were deemed to be significantly enriched if the adjusted *P* < 0.05 and the gene count in each GO term was more than 3.

### Functional Annotation of lncRNAs

The biological function of lncRNAs was annotated by overrepresentation enrichment analysis (ORA) of co-expressed protein-coding genes (PCGs). The PCGs were defined as co-expressed genes with a given lncRNA if the *P* < 0.0001 for the correlation coefficient test.

### Identification of Cis-Acting lncRNAs

As the lncRNAs may regulate the expression levels of the corresponding adjacent PCGs by cis-acting manner, the cis-acting lncRNAs were identified if the lncRNA and its adjacent PCGs (within one million base pairs) exhibit highly correlated expression (Pearson correlation coefficient > 0.5 or < −0.5).

## Results

### RNA Sequencing Method Reveals Diverse RNAs in Both AF and Control Groups

**Figure 1:**
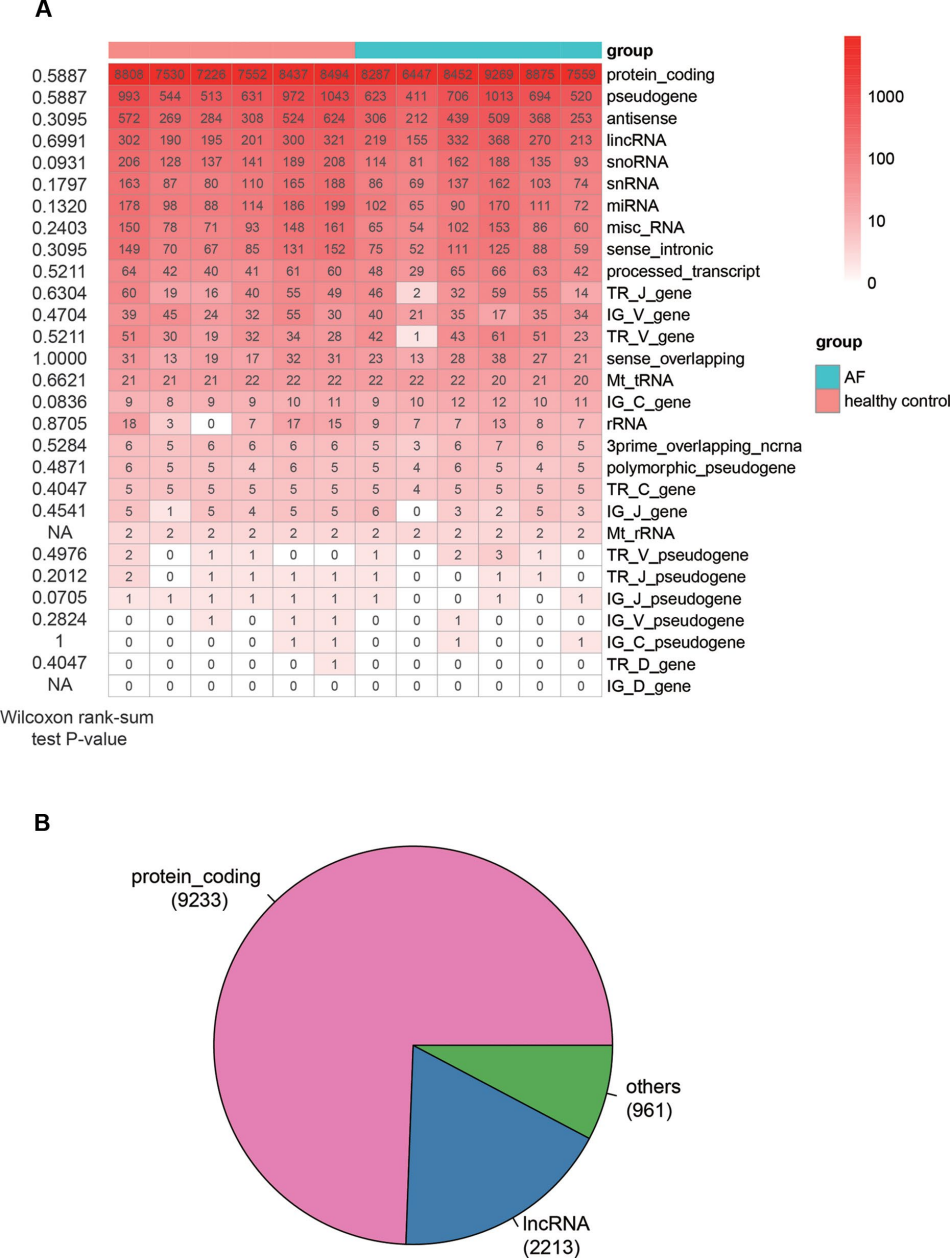
The overview of genes identified by RNA sequencing method. **(A)** The number of genes identified in each sample (FPKM > 1). The Wilcoxon rank-sum test *P*-values that compare the gene counts in AF with those in normal controls are listed on the left of the heat-map. **(B)** The pie chart displays the number of genes, including PCGs, lncRNAs, and other ncRNAs.

The analysis of sequencing data with 6 AF patients and 6 controls allowed us to identify 15,147 genes in total (FPKM > 1 in at least one sample), which consisted of 29 RNA categories, including protein-coding genes (PCGs), pseudogenes, antisense RNAs and etc. (**Figure 1A**). The PCGs, pseudogenes, and antisense RNAs accounted for about 90% of the total identified RNAs. For each RNA category, the number of genes in AF was not observed to be higher or lower than that in control (Wilcoxon rank-sum test, *P* < 0.05). In addition, we also considered genes with seven specific biotypes as lncRNAs (See Material and Methods). Given a threshold of FPKM >1 in at least 25% samples (n = 3), we identified 9,233 PCGs, 2,213 lncRNAs, and 961 other ncRNA genes, which were then used for downstream analysis (**Figure 1B**).

### Identification of Dysregulated mRNAs and lncRNAs in AF

**Figure 2:**
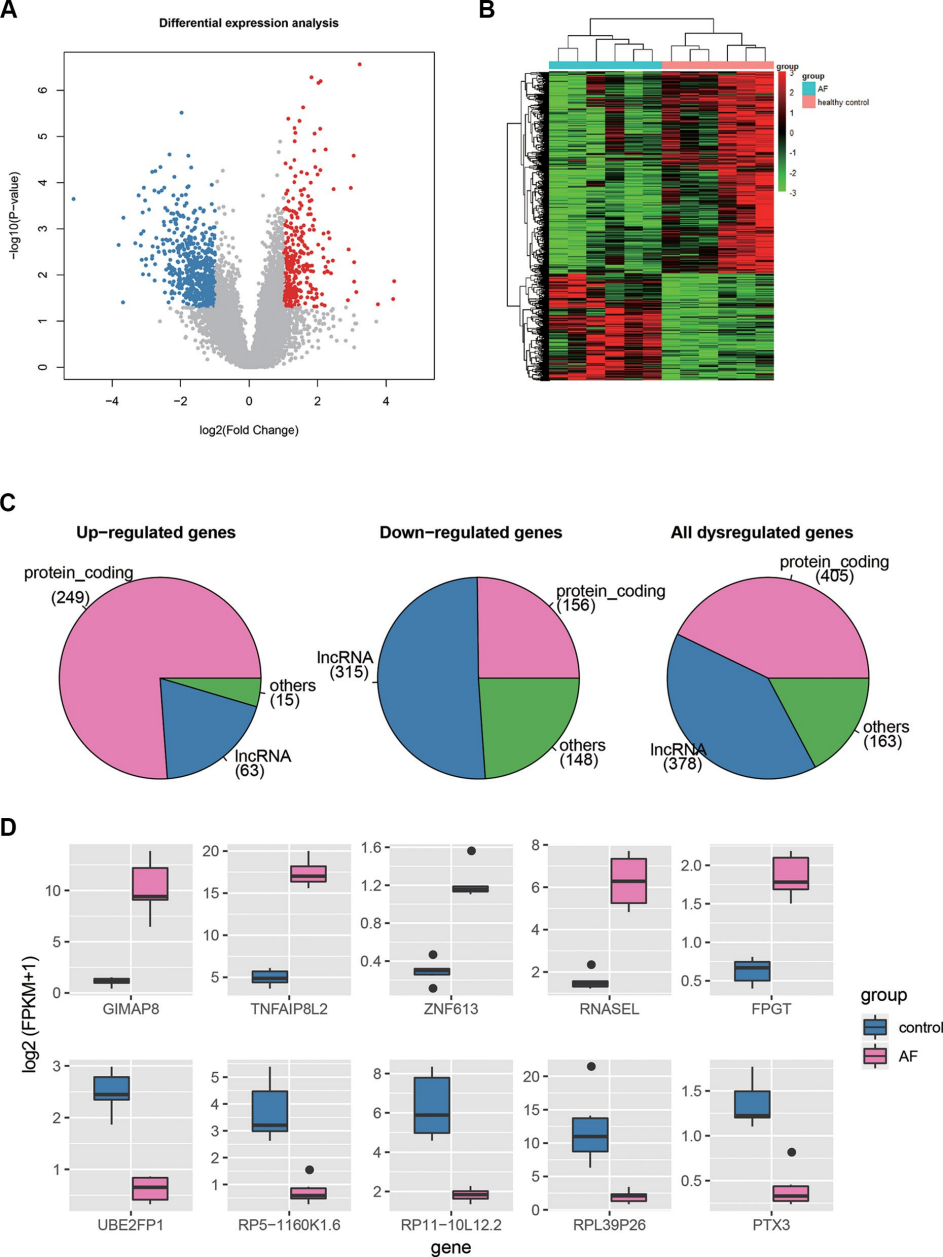
The differentially expressed genes in AF. **(A)** The volcano plot displays the up-regulated (red dots) and down-regulated (blue dots) genes. **(B)** The heat-map shows the scaled gene expression of dysregulated genes. **(C)** The number of PCGs, lncRNAs, and other ncRNAs in up-regulated, down-regulated, and all dysregulated genes. **(D)** The expression levels of the top-five up-regulated and down-regulated genes in AF and control.

Differential expression analysis was conducted to identify dysregulated genes in AF using the gene expression profiles. Specifically, a total of 946 genes, including 327 up- and 619 down-regulated genes, were differentially expressed in AF as compared with the healthy controls (t-test, *P* < 0.05 and fold change >2 or <^1^/_2_, **Figure 2A**, **Supplementary Table S1**). To investigate the distinction of the dysregulated genes between AF and healthy controls, we performed hierarchical clustering analysis of the dysregulated gene expression profiles, and found that the samples with AF could be clearly distinguished from the healthy controls (**Figure 2B**), indicating that gene expression profiles between AF and healthy controls had marked differences. Among the dysregulated genes, the proportion of PCGs was significantly higher in the up-regulated genes than in the down-regulated genes (**Figure 2C**, 249/327 vs. 156/619, proportion test, *P* < 0.0001). In contrast, the proportion of lncRNAs was observed higher in the down-regulated genes than in the up-regulated genes (**Figure 2C**, 315/619 vs. 63/327, proportion test, *P* < 0.0001).

Furthermore, we selected the top-five up- and down-regulated genes (**Figure 2D**), and found that the top-five up-regulated genes were all PCGs, while only one down-regulated gene encoded protein. Notably, three of the top-five upregulated genes, *GIMAP8*, *TNFAIP8L2*, and *RNASEL*, were involved in inflammatory response, suggesting that the dysregulation of inflammatory response may be the an important indicator for AF. On the other hand, the *PTX3* had an antiangiogenic role, and its downregulation may lead to enhanced angiogenesis. In addition, lncRNAs, HOTAIRM1, RP11-262H14.1, and RP11-84A19.4, have been reported to be dysregulated in AF by previous studies (Yu et al., 2017; Qian et al., 2019). These results indicated that differential expression analysis could uncover some key genes in AF.

### Gene Ontology-Based Enrichment Analysis of the Dysregulated Genes

**Figure 3:**
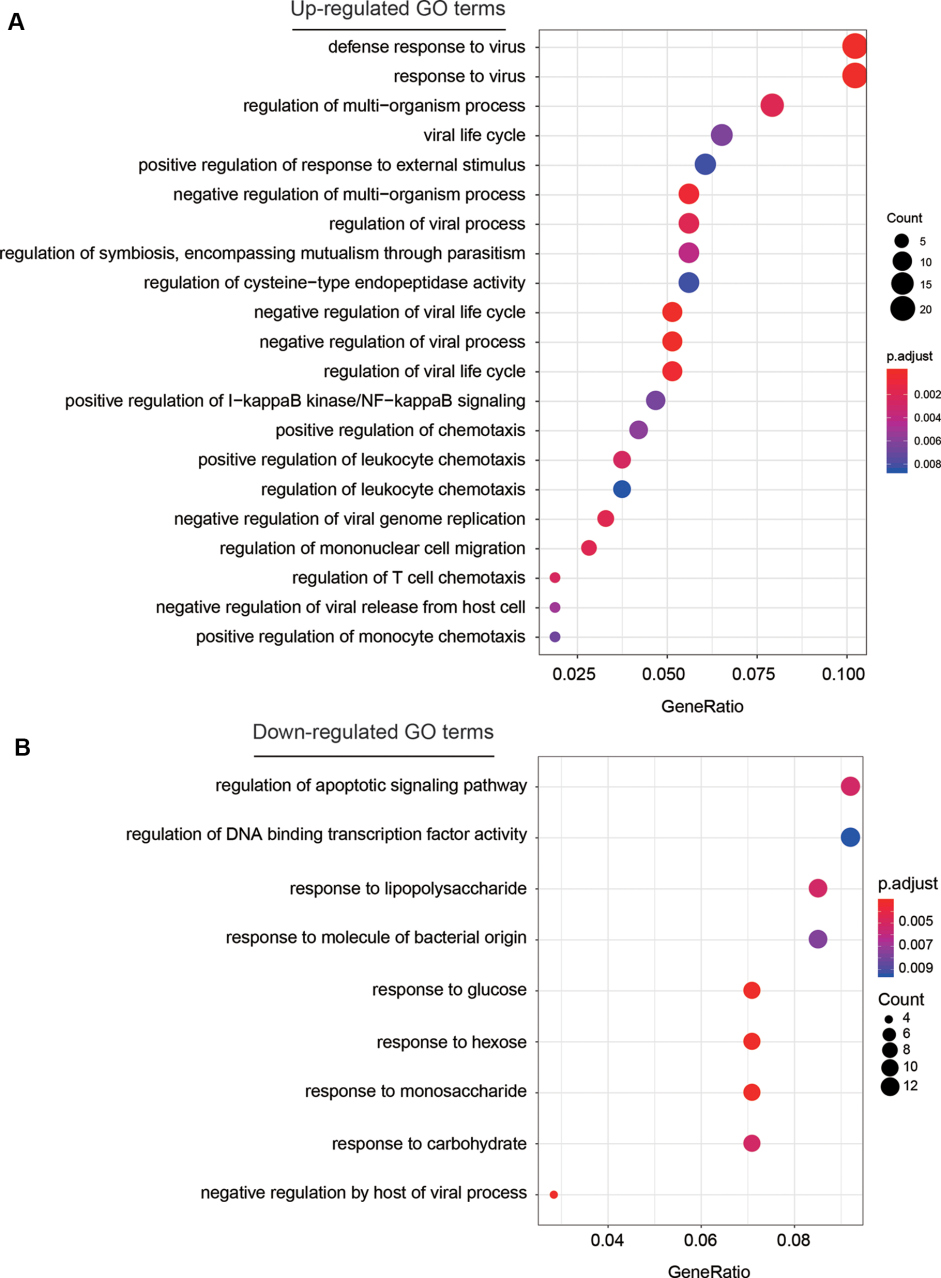
The GO terms enriched by dysregulated genes. The GO terms enriched by up-regulated and down-regulated genes are displayed in **(A)** and **(B)**, respectively. The more the gene count, the larger size the circle.

To investigate some key biological functions involved in AF, we performed overrepresentation enrichment analysis (ORA) on the up- and down-regulated genes, respectively. We found that the up-regulated genes were highly enriched in biological functions related to response to virus, such as defense response to virus, response to virus, viral life cycle, regulation of viral process, and regulation of viral life cycle, and related to inflammatory response, such as positive regulation of I-kappaB kinase/NF-kappaB signaling, and regulation of chemotaxis (**Figure 3A**, adjusted *P* < 0.05). These results indicated that chronic viral infection may lead to activated inflammatory pathways, thereby alter the electrophysiology, structure, and autonomic remodeling of the atria (Chiang et al., 2013).

Among the down-regulated GO terms, biological functions related to the response to saccharides (**Figure 3B**), such as response to lipopolysaccharide, response to glucose, response to hexose, response to monosaccharide, and response to carbohydrate were significantly enriched by the down-regulated genes. Notably, the weakened response to glucose in blood may reduce the insulin level, thereby lead to hyperglycemia, which further demonstrate the close association between hyperglycemia and AF (Rigalleau et al., 2002).

### Prediction of lncRNAs Regulating Expression of the Adjacent PCGs

**Figure 4:**
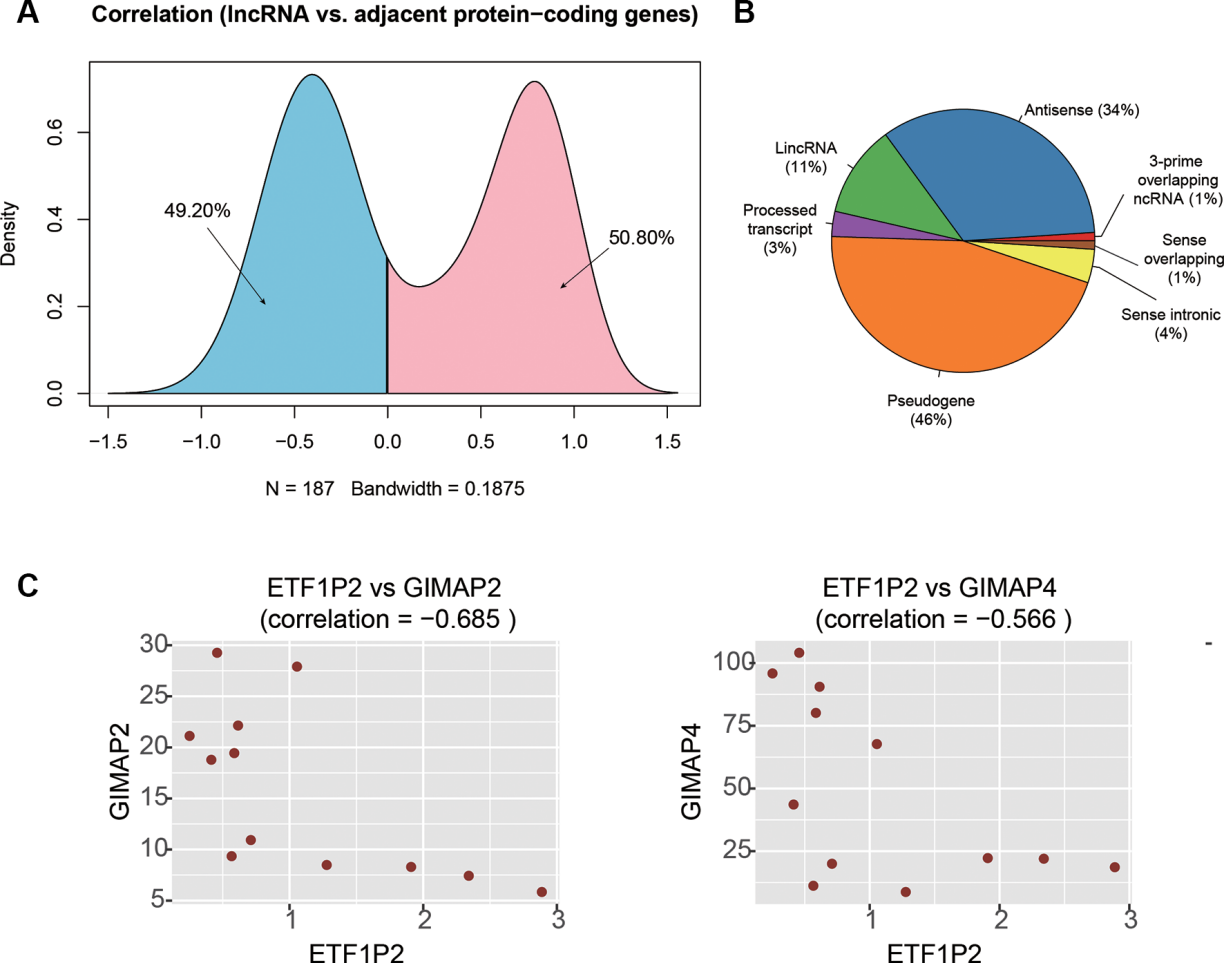
The cis-acting lncRNA candidates involved in AF. **(A)** The density of the correlation coefficients between lncRNAs and the corresponding adjacent PCGs. **(B)** The distribution of RNA biotypes for the cis-acting lnRNA candidates. **(C)** The correlation coefficients between ETF1P2 and GIMAP2, and between ETF1P2 and GIMAP4.

It has been widely recognized that lncRNAs could regulate the expression of the adjacent PCGs by cis-acting manner (Kornienko et al., 2013). To identify these cis-acting lncRNAs, we first searched the adjacent dysregulated PCGs within one million base pairs for each dysregulated lncRNA, and found 187 lncRNA-PCG pairs. The expression levels between the lncRNA and its corresponding PCGs were highly correlated (**Figure 4A**). Particularly, the expression levels of about half of the lncRNA-PCG pairs were negatively correlated, indicating that the lncRNAs may suppress the expression of the adjacent PCGs. With a threshold at Pearson correlation coefficient > 0.5 or < −0.5, we identified 71 lncRNA-PCG pairs, composed of 58 PCGs and 63 lncRNAs, with potential regulatory relationship (**Supplementary Table S2**). Among the cis-acting lncRNAs, we found that pseudogene (46%), antisense (34%), and lincRNA (11%) were the major categories (**Figure 4B**).

To identify key lncRNAs involved in regulating gene expression, we selected seven lncRNAs, *AL021707.2*, *CTD-2622I13.3*, *ETF1P2*, *RP11-4K3__A.5*, *RP11-95J11.1*, *ZNF137P*, and *H2AFZP1*, that regulated multiple PCGs. Notably, we found that *ETF1P2*, a pseudogene locating within 7q36, was negatively correlated with two adjacent PCGs with similar functions, *GIMAP2* and *GIMAP4* (**Figure 4C**), which participated in the regulation of T helper cell differentiation (Filen et al., 2009), indicating that the pseudogene *ETF1P2* may be the upstream regulator of T helper cell differentiation.

### Functional Annotation of the Dysregulated lncRNAs by Co-Expressed PCGs

**Figure 5:**
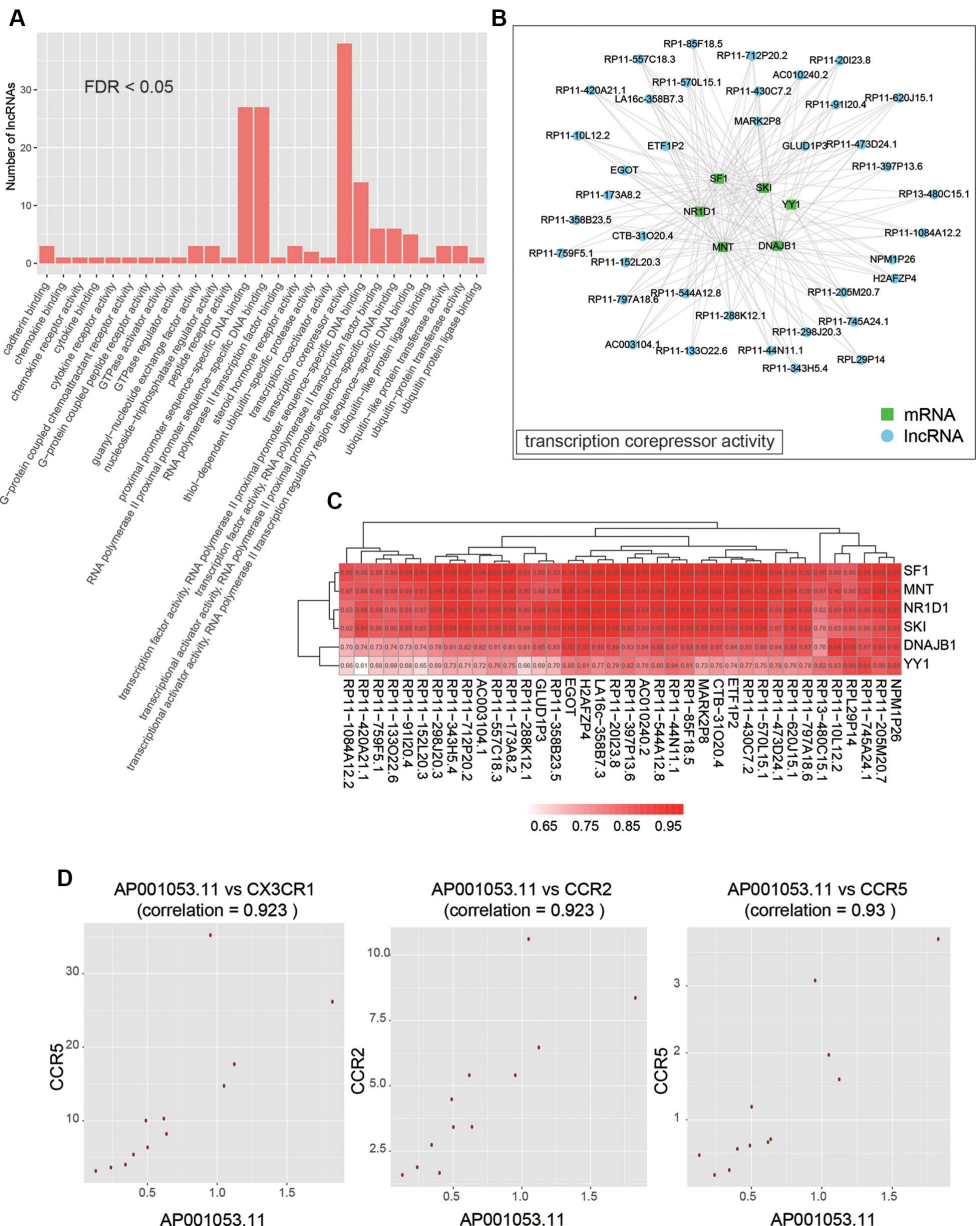
The functional annotation of lncRNAs by co-expressed PCGs. **(A)** The overview of the GO terms for the annotation of dysregulated lncRNAs (FDR < 0.05). **(B)** The PCGs and lncRNAs involved in transcription corepressor activity. **(C)** The correlation coefficients between PCGs and lncRNAs involved in transcription corepressor activity. **(D)** The scatter plots display the correlation between AP001053.11 and one of three chemokine receptors, including CX3CR1, CCR2, and CCR5.

As co-expressed genes are more likely to be co-regulated, sharing similar functions, or involved in similar biological processes (Stuart et al., 2003), we predicted the function of lncRNAs by performing overrepresentation enrichment analysis on the co-expressed PCGs to identify enriched GO terms (**Supplementary Table S3**). We found that a large number of lncRNAs (n > 20) had the biological functions termed transcription corepressor activity, proximal promoter sequence-specific DNA binding, and RNA polymerase II proximal promoter sequence-specific DNA binding (**Figure 5A**). Specifically, 38 lncRNAs were characterized with transcription corepressor activity, and highly correlated with five PCGs (Pearson correlation coefficient > 0.5), *SF1*, *MNT*, *NR1D1*, *SKI*, *DNAJB1*, and *YY1*, with the same GO term (**Figures 5B, C**). In addition, we also found that one lncRNA, *AP001053.11*, may participate in inflammatory response related GO terms, such as chemokine binding, chemokine receptor activity, cytokine binding, and cytokine receptor activity (**Figure 5D**). Notably, three chemokine receptor, *CX3CR1*, *CCR2*, and *CCR5*, were highly correlated with *AP001053.11* (Pearson correlation coefficient > 0.9), further suggesting a critical role of *AP001053.11* in regulation of chemokine receptor activity.

## Discussion

LncRNAs are an emerging class of RNA species that may play a critical regulatory role in gene expression. LncRNAs can serve as diagnostic biomarkers or therapeutic targets for many diseases (Ishii et al., 2006; Chen et al., 2008; Chubb et al., 2008). However, the association between lncRNAs and AF is still not fully understood.

In this study, we used RNA sequencing data to identify and quantify the both PCGs and lncRNAs, and conducted differential expression analysis to identify dysregulated genes in AF. Specifically, a total of 946 genes, including 327 up- and 619 down-regulated genes, were differentially expressed in AF as compared with the healthy controls (t-test, *P* <0.05 and fold change >2 or <^1^/_2_, **Figure 2A**, **Supplementary Table S1**). The hierarchical clustering analysis of those dysregulated gene expression profiles showed that the samples with AF could be clearly distinguished from the healthy controls (**Figure 2B**), indicating that gene expression profiles between AF and healthy controls had marked differences. Furthermore, we found that three of the top-five upregulated genes, *GIMAP8*, *TNFAIP8L2*, and *RNASEL*, were involved in inflammatory response, which was in accordance with the conclusion that the infiltration of immune cells and proteins that mediate inflammatory response in cardiac tissue and circulatory processes is associated with AF by previous studies (Yamashita et al., 2010; Harada et al., 2015). On the other hand, the *PTX3* had an antiangiogenic role, and its downregulation may lead to enhanced angiogenesis, which has been reported to be associated with AF (Berntsson et al., 2019).

To investigate some key biological functions involved in AF, we performed ORA on the dysregulated genes. The significant enrichment of these up-regulated genes in biological functions related to response to virus and inflammatory response suggested that chronic viral infection may lead to activated inflammatory pathways, thereby alter the electrophysiology, structure, and autonomic remodeling of the atria (Chiang et al., 2013). In contrast, the downregulated GO terms were related to the response to saccharides (**Figure 3B**), which gave us a hint that the weakened response to glucose in blood may reduce the insulin level, thereby lead to hyperglycemia as previous study reported (Rigalleau et al., 2002).

To identify key lncRNAs involved in AF, we predicted lncRNA-regulated expression of the adjacent PCGs, and characterized biological function of the dysregulated lncRNAs. We found that *ETF1P2*, a pseudogene locating within 7q36, was negatively correlated with two adjacent PCGs with similar functions, *GIMAP2* and *GIMAP4* (**Figure 4C**), which participated in regulation of T helper cell differentiation (Filen et al., 2009), indicating that the pseudogene *ETF1P2* may be an upstream regulator of T helper cell differentiation. Moreover, we also found that one lncRNA, *AP001053.11*, may participate in inflammatory-response-related GO terms by co-expression-based functional annotation. Notably, three chemokine receptor, *CX3CR1*, *CCR2*, and *CCR5*, were highly correlated with *AP001053.11* (Pearson correlation coefficient > 0.9), further suggesting that *AP001053.11* may be implicated in AF *via* the regulation of chemokine receptor activity.

In addition, there are also some limitations in this study. Firstly, more samples were needed to support our findings about the key lncRNAs. We will collect more samples with AF and healthy donors in the near future, which can overcome this limitation. Secondly, some experimental validation would be required for future verification of the functional lncRNAs. We hope to conduct further research with larger sample size, experimental validation and improved methodology for data analysis in the near future.

In conclusion, we have identified key PCGs and lncRNAs, which may be implicated in AF, which not only improves our understanding of the roles of lncRNAs in AF, but also provides potentially functional lncRNAs for AF researchers.

## Data Availability Statement

The datasets generated for this study can be found in the SRP093226.

## Author Contributions

Conception and design: D-MW, JL, G-QC, X-WH, Y-LZ; Administrative support: JL, G-QC, Y-LZ; Provision of study materials or patients: D-MW, S-HF, Z-HZ; Collection and assembly of data: D-MW, S-HF, XW, X-RH, SW, Y-JW, Z-FZ; Data analysis and interpretation: D-MW, QS, M-QL; Manuscript writing: All authors; Final approval of manuscript: All authors.

## Funding

This work was supported by the Priority Academic Program Development of Jiangsu Higher Education Institutions (PAPD); the 2016 “333 Project” Award of Jiangsu Province, the 2013 “Qinglan Project” of the Young and Middle-aged Academic Leader of Jiangsu College and University, the National Natural Science Foundation of China (81871249, 81571055, 81400902, 81271225, 81171012, and 30950031), the Major Fundamental Research Program of the Natural Science Foundation of the Jiangsu Higher Education Institutions of China (13KJA180001), and grants from the Cultivate National Science Fund for Distinguished Young Scholars of Jiangsu Normal University.

## Conflict of Interest

The authors declare that the research was conducted in the absence of any commercial or financial relationships that could be construed as a potential conflict of interest.
